# MUC1 ectodomain is a flagellin-targeting decoy receptor and biomarker operative during *Pseudomonas aeruginosa* lung infection

**DOI:** 10.1038/s41598-021-02242-x

**Published:** 2021-11-22

**Authors:** Avelino C. Verceles, Pavan Bhat, Zain Nagaria, Destiny Martin, Harsh Patel, Afua Ntem-Mensah, Sang W. Hyun, Andrea Hahn, Jean Jeudy, Alan S. Cross, Erik P. Lillehoj, Simeon E. Goldblum

**Affiliations:** 1 Department of Medicine, University of Maryland School of Medicine, Baltimore, MD USA; 2 U.S. Department of Veterans Affairs, Baltimore VA Medical Center, Baltimore, MD USA; 3 Division of Infectious Diseases, Children’s National Health System, Washington, DC USA; 4 Department of Pediatrics, George Washington University School of Medicine and Health Sciences, Washington, DC USA; 5 Department of Diagnostic Radiology and Nuclear Medicine, University of Maryland School of Medicine, Baltimore, MD USA; 6 Center for Vaccine Development and Global Health, University of Maryland School of Medicine, Baltimore, MD USA; 7 Department of Pediatrics, University of Maryland School of Medicine, Baltimore, MD USA

**Keywords:** Microbiology, Diseases, Medical research, Pathogenesis

## Abstract

We previously reported that flagellin-expressing *Pseudomonas aeruginosa* (Pa) provokes NEU1 sialidase-mediated MUC1 ectodomain (MUC1-ED) desialylation and MUC1-ED shedding from murine lungs in vivo. Here, we asked whether Pa in the lungs of patients with ventilator-associated pneumonia might also increase MUC1-ED shedding. The levels of MUC1-ED and Pa-expressed flagellin were dramatically elevated in bronchoalveolar lavage fluid (BALF) harvested from Pa-infected patients, and each flagellin level, in turn, predicted MUC1-ED shedding in the same patient. Desialylated MUC1-ED was only detected in BALF of Pa-infected patients. Clinical Pa strains increased MUC1-ED shedding from cultured human alveolar epithelia, and FlaA and FlaB flagellin-expressing strains provoked comparable levels of MUC1-ED shedding. A flagellin-deficient isogenic mutant generated dramatically reduced MUC1-ED shedding compared with the flagellin-expressing wild-type strain, and purified FlaA and FlaB recapitulated the effect of intact bacteria. Pa:MUC1-ED complexes were detected in the supernatants of alveolar epithelia exposed to wild-type Pa, but not to the flagellin-deficient Pa strain. Finally, human recombinant MUC1-ED dose-dependently disrupted multiple flagellin-driven processes, including Pa motility, Pa biofilm formation, and Pa adhesion to human alveolar epithelia, while enhancing human neutrophil-mediated Pa phagocytosis. Therefore, shed desialylated MUC1-ED functions as a novel flagellin-targeting, Pa-responsive decoy receptor that participates in the host response to Pa at the airway epithelial surface.

## Introduction

Ventilator-associated pneumonia (VAP) is defined by the appearance of a new pulmonary infiltrate of infectious origin occurring more than 48 h after endotracheal (ET) intubation^1^. This common nosocomial infection occurs in approximately 10% of ventilated patients^2^. Although the reported incidences per 1000 ventilator days has decreased from 3.1 to 0.9 in medical intensive care units (ICUs), and from 5.2 to 2.0 in surgical ICUs, attributable morbidity and mortality remain relatively high^3,4^. All-cause mortality associated with VAP is reportedly 20% to 50%, while attributable mortality is estimated to be 13%^1,5^. VAP is associated with prolonged hospitalization and increased cost. In risk-matched patients either with or without VAP, the presence of pneumonia added an average of US $40,000 to hospital costs and 10 days to total time on mechanical ventilation, ICU stay, and/or overall hospitalization^6^. Prolonged intubation is a primary driver of infection, with risk of VAP increasing by 2% per day, starting in the second week post-intubation^7^. Other modifiable risk factors include the use of antacids, supine position, and receipt of enteral nutrition. Non-modifiable risk factors include coma, age greater than 60 years, head trauma, chronic obstructive pulmonary disease, and male gender. Intervention-related factors, such as neurologic or thoracic surgery and reintubation, contribute to VAP as well^7^.

For the diagnosis of VAP, the findings of a new pulmonary infiltrate in the setting of fever, purulent sputum, leukocytosis, and hypoxemia provide a reported sensitivity of 69% and specificity of 75%^1^. This translates to positive and negative likelihood ratios of 2.5 and 0.06, respectively^8^. However, no known combination of signs and symptoms provides a definitive diagnosis. Addition of noninvasive, semiquantitative sputum cultures by ET aspirate, can help with organism- and susceptibility-directed therapy^1^. In these critically-ill patients, the absence of a reliable diagnostic approach can lead to over-diagnosis and overuse of broad-spectrum antibiotics, encouraging the development of multidrug-resistant (MDR) pathogens^9^.

The predominant mechanisms underlying VAP pathogenesis include microaspiration of oropharyngeal secretions and bacterial biofilm formation within the ET tube^10^. Whether pneumonia ensues is dictated by organism virulence factors, inoculum size, and the host immune response^11^. In the ventilated patient, the presence of an ET tube impairs the cough reflex, decreasing the ability to expel pathogens. Over time, the ET tube luminal surface serves as a nidus for bacterial colonization and biofilm formation that can be impregnable to antibiotics. These bacteria can then be introduced into the lower respiratory tract by positive ventilatory pressure^10^.

*Pseudomonas aeruginosa* (Pa), a flagellated, Gram-negative bacillus, is a leading pathogen implicated in VAP, with a reported frequency approaching 25%^12^. Nonventilated patients who are debilitated with burns, neutropenia, or cystic fibrosis are also at increased risk of Pa acquisition^13^. Pa commonly colonizes healthcare workers, and in one study, was recovered from 22% of hospital rooms^8^. Hospital reservoirs include showers, cleaning equipment, bronchoscopes, endoscopes and respiratory therapy equipment^13^. Antibacterial-susceptible strains that colonize patients can become MDR through ubiquitous antibiotic use and selective pressure.

The first host barrier with which Pa interacts in the airways is the mucus blanket which covers the epithelium^14,15^. This mucus blanket made up of two distinct yet interacting layers, a highly viscous gel layer overlying a periciliary liquid layer. Members of the mucin family comprise the major protein component of mucus and are divided into secreted and cell-associated mucins. Of the 5 major mucins that have been detected in the lung, two are secreted, MUC5AC and MUC5B, and three are cell-tethered, MUC1, MUC4, and MUC16. MUC1 is comprised of an NH_2_-terminal MUC1 ectodomain (MUC1-ED) coupled to a COOH-terminal MUC1 cytoplasmic domain (MUC1-CD)^14,15^. The MUC1-ED contains a variable number of highly sialylated, 20-amino acid (aa) tandem repeats distal to a juxtamembranous glycine-serine protease recognition site. The proline-rich MUC1-ED exhibits an extended, rod-like conformation that protrudes higher above the EC surface than other membrane-associated proteins^16^. Here, the MUC1-ED is strategically positioned to interact with flagellin-expressing Pa in the airway lumen. The MUC1-ED is up to 90% carbohydrate, almost entirely through more than 500 predicted O-linked glycosylation sites within its tandem repeats^17^. The relatively short, 72-aa MUC1-CD contains multiple binding sites for cytosolic signaling molecules^18–20^.

**Figure 1:**
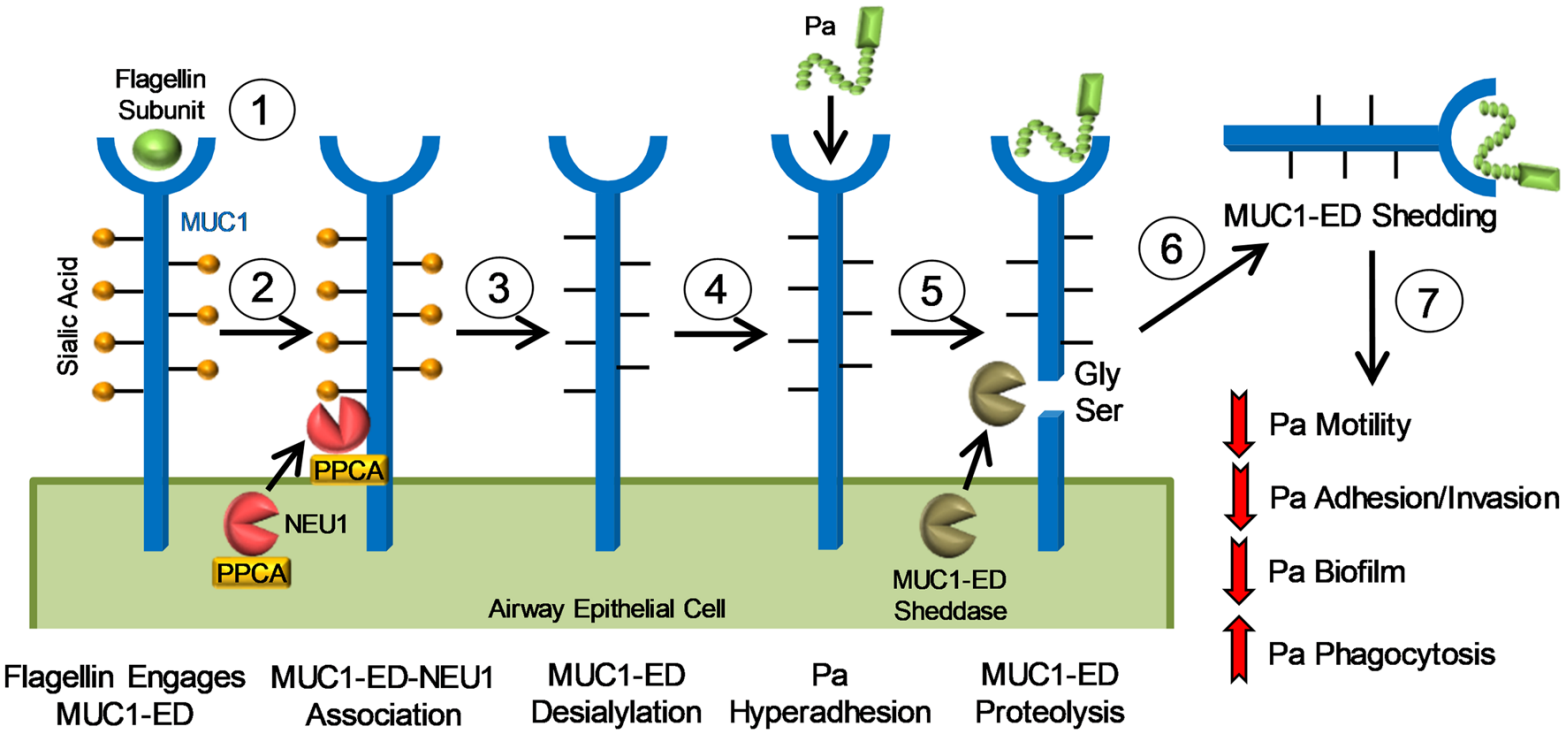
Pa-expressed flagellin provokes NEU1-mediated MUC1-ED desialylation to generate a flagellin-targeting MUC1-ED decoy receptor. The Pa flagellin subunit engages cell-associated MUC1-ED (step 1), leading to recruitment of a preformed pool of intracellular NEU1, together with its chaperone protein, PPCA, to MUC1-ED (step 2). NEU1 desialylates the MUC1-ED (step 3), to increase its adhesiveness for Pa (step 4) and unmask its glycine-serine (Gly-Ser) protease recognition site (step 5), permitting sheddase-mediated MUC1-ED release from the airway EC surface (step 6). Shed, desialylated MUC1-ED in the airway lumen acts as a soluble decoy receptor that reduces Pa motility, competitively inhibits Pa adhesion to cell-associated MUC1-ED and subsequent invasion of airway epithelia, protects against Pa biofilm formation, and enhances Pa phagocytosis by host PMNs (step 7).

We had previously established that Pa-expressed flagellin engages the MUC1-ED expressed on the surface of airway epithelial cells (ECs)^21^ (Fig. 1). We found that this receptor-ligand interaction recruits the eukaryotic sialidase, NEU1, together with its chaperone protein, protective protein/cathepsin A (PPCA), to surface-expressed MUC1^21,22^. Further, we found that NEU1 desialylates the MUC1-ED to unmask cryptic binding sites for flagellin-expressing Pa^21,23^ (Fig. 1). In contrast, NEU1-mediated MUC1-ED desialylation did not increase its adhesiveness for a flagellin-deficient, isogenic Pa mutant^21^. While NEU1 overexpression in MUC1-expressing human embryonic kidney (HEK)293 cells enhanced their adhesiveness for wild-type Pa, NEU1 overexpression in Toll-like receptor (TLR)5-expressing HEK293 cells did not^21^. Although NEUs are well established virulence factors for multiple bacterial pathogens^24^, this is the only case, to our knowledge, where a prokaryote hijacks a host sialidase to influence its pathogenicity. At the same time, we also found that NEU1-mediated MUC1-ED desialylation uncovers a juxtramembranous protease recognition site, permitting MUC1-ED cleavage by endogenous sheddases and release from the EC surface^21,25–27^ (Fig. 1). More specifically, NEU1 overexpression in HEK293 cells expressing a MUC1-ED S317A mutant, containing a serine-to-alanine substitution within the glycine-serine protease recognition site, displayed no MUC1-ED shedding^22^. Shed, desialylated MUC1-ED in the airway lumen retains its ability to bind to flagellin-expressing Pa, thereby acting as a soluble decoy receptor that reduces Pa motility and competitively inhibits Pa adhesion to cell-associated MUC1-ED^21,22^, both reducing Pa invasion of underlying tissues^22^. In fact, in a mouse model of Pa pneumonia, co-administration of the MUC1-ED decoy receptor with a lethal challenge of Pa diminished lung bacterial burden, lung cytokine production, and pulmonary leukostasis, and increased 5-day survival from 0 to 77%, indicating that the shed MUC1-ED constitutes a protective component of the host response to Pa^22^ (Fig. 1). In the current studies, we sought to extend our prior findings in the mouse model to an in vivo human setting. We asked whether the shed MUC1-ED levels might be elevated in BALF of Pa-infected patients, as an indirect but specific measure of NEU1 catalytic activity^21,23^. Further, we tested human recombinant MUC1-ED for its ability to influence Pa adhesion to alveolar epithelia, Pa motility, Pa biofilm production, Pa-stimulated interleukin-8 (IL-8) production, and neutrophil-mediated Pa phagocytosis.

## Results

### MUC1-ED levels in bronchoalveolar lavage fluid (BALF) from Pa-infected patients

We previously reported that Pa increases MUC1-ED shedding using an in vivo mouse model of Pa pneumonia^22^. We asked whether MUC1-ED levels also might be increased in BALF from patients with Pa airway infection. Between 12 June 2013 and 20 February 2020, BALF was collected from 61 patients undergoing mechanical ventilation and clinically diagnosed with VAP in three separate units at the University of Maryland Medical Center (Baltimore, MD). Of these 61 patients, the BALF of 13 (21.3%) were culture-positive for Pa, 32 (52.5%) culture-positive for other microorganisms, 6 of which were polymicrobial, and 16 (26.2%) culture-negative (Table 1). Power analysis indicated that this study is 100% powered with these sample sizes to detect different BALF MUC1-ED levels in Pa-positive (n = 13) *vs*. Pa-negative (n = 48) patients. Pa-infected VAP patients were more likely to have been admitted to the long-term Comprehensive Pulmonary Rehabilitation Unit (CPRU) (61.5% *vs*. 12.5%), and experienced fewer ventilator-free days (8.2 *vs*. 18.7 days), compared with Pa-negative patients (Table 1). Interestingly, Pa-infected VAP patients were less likely to have been admitted to the acute care Medical Intensive Care Unit (MICU) compared with Pa-negative patients (38.5% *vs*. 81.2%). None of the other clinical/demographic parameters measured were different between Pa-infected patients and Pa-negative patients.

**Table 1:** Patient characteristics.

VAP Patients	*P*. aeruginosa (*n* = 13)	Other microorganisms (n = 32)^a^ or culture-negative (n = 16)	*p* Value
Age in years, mean S.D	61.8 ± 9.8	58.6 ± 12.7	0.42
BMI, mean S.D	32.1 ± 11.9	31.3 ± 10.6	0.81
Ventilator-free days, mean S.D	8.2 ± 11.3	18.7 ± 10.0	0.002
APACHE II Score, mean ± S.D	17.7 ± 7.0	18.6 ± 8.7	0.72
**Race, n**			
White	7 (53.8%)	27 (56.2%)	0.88
African American	6 (46.2%)	18 (37.5%)	0.58
Other	0 (0.0%)	3 (6.3%)	0.36
**Gender, n**			
Male	4 (30.8%)	24 (50.0%)	0.22
Female	9 (69.2%)	24 (50.0%)	0.22
**ICU Locationm, n**			
Acute Care MICU	5 (38.5%)	39 (81.2%)	0.002
Acute Care CCRU	0 (0.0%)	3 (6.3%)	0.36
Long-Term CPRU	8 (61.5%)	6 (12.5%)	< 0.001
Antibiotics, n	13 (100%)	46 (96.0%)	0.45
Anti-Pseudomonal Antibiotics	13 (100%)	39 (81.3%)	0.11
Chest X-ray Abnormality, n	13 (100%)	46 (96.0%)	0.45

^a^*Aspergillus* spp. (n = 1), *Candida* spp. (n = 2), *Candida albicans* (n = 5), *Candida parapsilosis* (n = 1), *Enterococcus* spp. (n = 4), *Escherichia coli* (n = 1), *Klebsiella oxytoca* (n = 1), *Klebsiella pneumoniae* (n = 1), *Lactobacillus* spp. (n = 1), *Mycobacterium chelonae* (n = 1), normal respiratory flora (n = 4), *Proteus mirabilis* (n = 1), *Rothia* spp. (n = 1), *Staphylococcus* spp. (n = 3), *Staphylococcus aureus* (n = 1), *Streptococcus* spp. (n = 6), *Streptococcus pyogenes* (n = 1).

**Figure 2:**
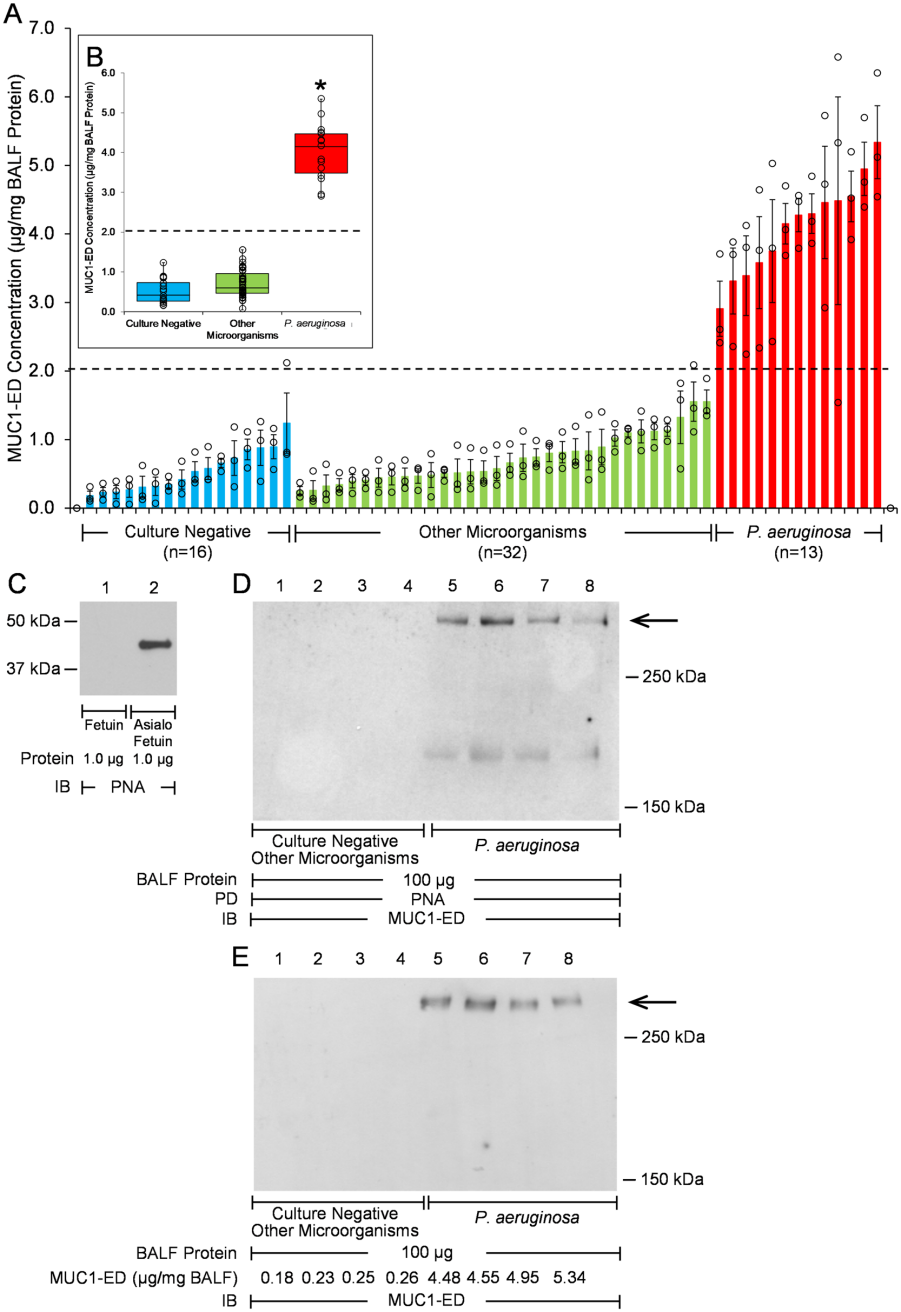
MUC1-ED levels are increased in BALF from *P. aeruginosa*-infected VAP patients. (**A**) MUC1-ED levels in BALF from noninfected patients (n = 16), patients infected with microorganisms other than Pa (n = 32), or Pa-infected patients (n = 13) were quantified by ELISA and normalized to total BALF protein. The dashed line represents an arbitrary cut-off value to differentiate BALF MUC1-ED levels in Pa-infected patients from patients infected with microorganisms other than Pa or noninfected patients. Bars represent mean ± S.E. values (n = 3). Open circles represent individual values. (**B**) Box and whisker plots of the data in (**A**). *, increased MUC1-ED levels compared with noninfected patients or patients infected with microorganisms other than Pa at p < 0.05. (**C**) Fetuin and asialofetuin (1.0 µg) were processed for PNA lectin blotting (lanes 1, 2) as controls to validate PNA specificity. (**D**, **E**) Equal protein aliquots (100 µg) of BALF from noninfected patients or patients infected with microorganisms other than Pa (lanes 1–4), or Pa-infected patients (lanes 5–8) were (**D**) incubated with PNA-agarose and the PNA-binding proteins processed for MUC1-ED immunoblotting or (**E**) directly processed for MUC1-ED immunoblotting. MUC1-ED concentrations by ELISA are indicated under each lane in (**E**). Molecular weights in kDa are indicated on the right. Each arrow indicates the > 250 kDa MUC1-ED band of interest. PD, pull down; IB, immunoblot. The full-length immunoblot is shown. The results are representative of 2 independent experiments.

MUC1-ED levels in these 61 BALF samples were quantified by enzyme-linked immunosorbent assay (ELISA) and normalized to total BALF protein. Mean ± S.E. normalized MUC1-ED levels were 4.01 ± 0.19 µg/mg BALF protein (range, 2.87 µg/mg to 5.34 µg/mg) in the BALF from the 13 Pa-infected VAP patients compared with 0.71 ± 0.05 µg/mg (range, 0.05 µg/mg to 1.55 µg/mg) in the BALF from the 32 patients infected with other microorganisms and 0.52 ± 0.08 µg/mg (range, 0.17 µg/mg to 1.24 µg/mg) in the BALF from the 16 culture-negative patients (Fig. 2A,B). Therefore, the mean normalized MUC1-ED level in Pa-infected VAP patients was 5.6-fold greater than that measured in patients infected with other microorganisms, and 7.7-fold greater compared with culture-negative patients. These combined data suggest that elevated BALF levels of MUC1-ED might provide a diagnostic biomarker for Pa lung infection. We asked whether MUC1-ED levels in BALF of Pa-infected VAP patients might correlate with Acute Physiology and Chronic Health Evaluation (APACHE) II scores as a predictor of disease severity. In 11 patients where APACHE II scores were available, MUC1-ED levels did not correlate with APACHE II values (Supplemental Fig. 1).

Since NEU1-mediated MUC1-ED desialylation is required for its proteolytic release and shedding^22^, we next asked whether MUC1-ED in BALF from Pa-infected patients was desialylated. MUC1-ED immunoblotting of BALF proteins bound to the lectin, peanut agglutinin (PNA), which binds to subterminal galactose residues only after removal of terminal sialic acid, was used to address this question. First, we validated the specificity of the PNA lectin. As anticipated, PNA recognized asialofetuin, but not sialylated fetuin (Fig. 2C). MUC1-ED in the BALF of Pa-infected VAP patients was desialylated, while desialylated MUC1-ED in the BALF from Pa-negative patients was not detected (Fig. 2D). Similarly, total shed MUC1-ED was detected in the BALF from Pa-infected VAP patients, but not in the BALF from Pa-negative patients (Fig. 2E). The total MUC1-ED concentrations in the four selected samples from the Pa-positive group were comparable, as were the MUC1-ED levels in the four selected Pa-negative samples (Fig. 2E). Of note, although low levels of MUC1-ED were seen by ELISA in BALF harvested from the two Pa-negative patient groups (Fig. 2A,B), desialylated MUC1-ED could not be detected in Pa-negative patients either by the combined PNA enrichment/MUC1-ED immunoblotting protocol (Fig. 2D, lanes 1–4) or the immunoblotting-only protocol (Fig. 2E, lanes 1–4). It is conceivable that the relatively higher sensitivity of the ELISA procedure, compared with the PNA/immunoblotting or immunoblotting-only protocols, allows detection of very low levels of MUC1-ED by the former and not the latter.

MUC1-ED levels were also quantified in tracheal aspirate (TA)s and BALFs simultaneously collected from a subset of VAP patients (n = 15), including 2 patients infected with Pa, 6 patients infected with other microorganisms, and 7 culture-negative patients. No correlation was observed between MUC1-ED levels in the paired BALF and TA samples (Supplemental Fig. 2). Therefore, the MUC1-ED levels in the more easily obtained TA samples did not reflect BALF levels. However, due to the small sample size for TAs obtained from patients who cultured positive for Pa, whether a correlation exists could not be definitively excluded.

### MUC1-ED shedding by Pa-derived flagellin

**Figure 3:**
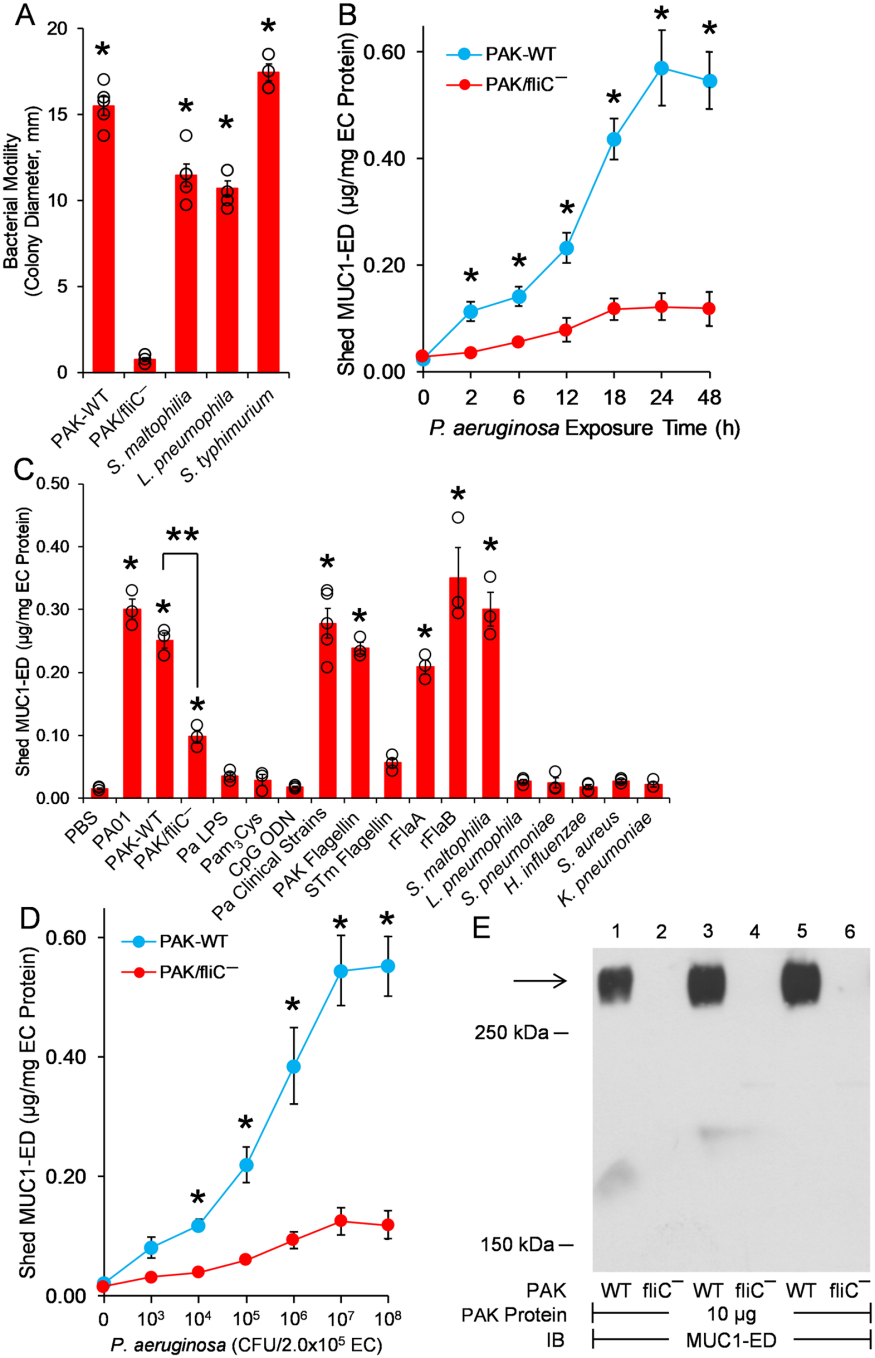
Flagellin-expressing Pa induces MUC1-ED shedding in vitro. (**A**) Bacterial motility assays were used to verify flagellin expression by *S. maltophilia*, *L. pneumophila*, and *S. enterica* serovar Typhimurium. WT-PAK and the PAK/fliC¯ flagellin-deficient strain were used as positive and negative controls, respectively. Bars represent mean ± S.E. colony diameters (mm) as a functional assay for motility (n = 5). *, increased motility compared with the PAK/fliC¯ strain at *p* < 0.05. (**B**) To establish the time profile for MUC1-ED shedding, A549 cells (2.0 × 10^5^ cells/well) cultured in 24-well plates were incubated for increasing times with fixed inocula of either WT-PAK or the PAK/fiC¯ mutant (1.0 × 10^7^ CFUs/well). At each time point, MUC1-ED levels in cell culture supernatants were measured by ELISA and normalized to total A549 cell protein. (**C**) A549 cells (2.0 × 10^5^ cells/well) in 24-well plates were incubated for 24 h at 37 °C with 1.0 × 10^7^ CFUs/well of the indicated bacterial strains, 100 ng/well of Pa LPS, 10 µg/well of Pam_3_Cys-Ser-(Lys)_4_, 10 µg/well of CpG ODN 1826, 10 ng/well of Pa or STm flagellins, 10 ng/well of Pa rFlaA or rFla flagellins, or the PBS vehicle control. MUC1-ED levels in cell culture supernatants were measured by ELISA and normalized to total A549 cell protein. (**D**) A549 cells (2.0 × 10^5^ cells/well) cultured in 24-well plates were incubated for 24 h at 37 °C with increasing CFUs/well of WT-PAK or the PAK/fliC¯ flagellin-deficient strain. MUC1-ED levels in cell culture supernatants were measured by ELISA and normalized to total cell protein. (**B**–**D**) Bars and data points represent mean ± S.E. values (n = 3 or 5). (**A**, **C**) Open circles represent individual values. *, increased MUC1-ED levels compared with (**A**, **B**, **D**) PAK/fliC¯ or (**C**) the PBS control at *p* < 0.05. **, decreased MUC1-ED levels provoked by the PAK/fliC¯ flagellin-deficient strain compared with WT-PAK at *p* < 0.05. (**E**) WT-PAK (lanes 1, 3, 5) or the PAK/fliC¯ mutant strain (lanes 2, 4, 6) were co-cultured for 24 h with A549 cells, the supernatants collected, and centrifuged at 5,000 xg for 10 min to collect the bacteria. The bacteria were lysed and equal protein aliquots (10 µg) of the lysates processed for MUC1-ED immunoblotting to detect Pa:MUC1-ED complexes. Molecular weights in kDa are indicated on the left. The arrow indicates the > 250 kDa MUC1-ED band of interest. IB, immunoblot. The full-length immunoblot is shown. The results are representative of 2 or 3 independent experiments.

MUC1-ED levels are increased in the BALF of mice challenged with either flagellin-expressing Pa or purified Pa-derived flagellin^22^ and in BALF from VAP patients infected with Pa (Fig. 2A,B). We asked whether Pa-derived flagellin might be responsible for MUC1-ED shedding, in vitro. First, we used an in vitro bacterial motility assay to establish flagella expression in selected microorganisms, including wild-type (WT)-PAK, *Stenotrophomonas maltophilia*, *Legionella pneumophila*, and *Salmonella enterica* serovar Typhimurium. A nonmotile PAK/fliC¯ flagellin-deficient, isogenic mutant strain was used as a negative control. With the exception of the PAK/fliC¯ strain, all microorganisms were motile (Fig. 3A). We next established the time requirements for WT-PAK-provoked increases in MUC1-ED shedding from in vitro cultures of A549 human alveolar epithelial cells (ECs) (Fig. 3B). At times ≥ 2 h, shed MUC1-ED levels were increased over time, with maximal levels at 24 h. Over the same time period, the PAK/fliC¯ mutant strain only induced minimal MUC1-ED shedding at later time points that did not achieve statistical significance compared with the phosphate-buffered saline (PBS) vehicle control. Stimulation of in vitro cultures of A549 ECs with either of two Pa laboratory strains, PA01 or PAK, dramatically increased MUC1-ED levels in cell culture supernatants compared with the PBS control (Fig. 3C). A549 EC stimulation with the PAK/fliC¯ flagellin-deficient strain was associated with dramatically reduced MUC1-ED shedding compared with that provoked by flagellin-expressing WT-PAK. However, the PAK/fliC¯ strain still increased MUC1-ED shedding compared with the PBS control, indicating that Pa constituent(s) other than flagellin might also be operative. Purified Pa lipopolysaccharide (LPS), Pam_3_Cys-Ser-(Lys)_4_ as a bacterial lipoprotein mimetic, and CpG oligodeoxynucleotide (ODN) 1826 as a bacterial DNA mimetic, each failed to increase MUC1-ED shedding (Fig. 3C). Whether other untested bacterial components contribute to Pa flagellin-provoked MUC1-ED shedding remains unclear. Five clinical strains of Pa isolated from blood cultures of pneumonia patients stimulated MUC1-ED shedding comparable with the PA01 and PAK laboratory strains. A549 cells were incubated with two flagellated and four nonflagellated respiratory pathogens. Of the two flagellated bacteria, *S. maltophilia* increased MUC1-ED shedding, whereas *L. pneumophila* did not (Fig. 3C). None of the four nonflagellated bacteria, including *Streptococcus pneumoniae*, *Haemophilus influenzae*, *Staphylococcus aureus*, or *Klebsiella pneumoniae*, increased MUC1-ED shedding (Fig. 3C). Each Pa strain expresses one of two types of flagellin, a-type (FlaA) or b-type (FlaB)^28^. Purified FlaA flagellin isolated from the PAK strain, and *Escherichia coli-*expressed recombinant (r)FlaA and rFlaB, cloned from PAK and PA01, respectively, stimulated MUC1-ED shedding (Fig. 3C). Since Pa-expressed flagellin clearly provoked MUC1-ED shedding, we asked whether the established pattern recognition receptor for bacterial flagellins, TLR5^29^, might be operative. To address this issue, we introduced a well-recognized TLR5 agonist, *S. enterica* serovar Typhimurium (STm)-derived flagellin^30^. STm-derived flagellin provoked far less MUC1-ED shedding than did Pa-derived flagellin, excluding participation of TLR5 (Fig. 3C). In other experiments, A549 ECs were stimulated with increasing inocula of WT-PAK or PAK/fliC¯, and after 24 h, cell culture supernatants collected, and MUC1-ED levels assayed and normalized to total EC protein. WT-PAK inocula ≥ 1.0 × 10^4^ colony forming units (CFUs) dose-dependently increased MUC1-ED shedding compared with an identical inoculum of PAK/fliC¯ (Fig. 3D). Therefore, flagellin-expressing Pa and *S. maltophilia*, as well as Pa-derived flagellin itself, each specifically induces MUC1-ED shedding from the surface of cultured human alveolar epithelia, in vitro.

### Formation of complexes comprised of flagellin-expressing Pa and the shed MUC1-ED decoy receptor

Since NEU1-mediated MUC1-ED desialylation both renders the ED hyperadhesive for Pa^21^ and unmasks its protease recognition site, permitting its proteolytic release^21,22^, we asked whether shed MUC1-ED might bind to flagellin-expressing Pa. WT-PAK and the PAK/fliC¯ strain each were co-cultured for 24 h with A549 cells, the supernatants collected and centrifuged to collect the bacteria. The collected Pa were lysed and the lysates processed for MUC1-ED immunoblotting (Fig. 3E). The WT-PAK was detected in Pa:MUC1-ED complexes, whereas the PAK/fliC¯ strain was not. Whether these Pa:MUC1-ED complexes form with cell-tethered MUC1-ED at the airway epithelial cell surface and are then released, and/or form with already shed MUC1-ED unoccupied by Pa, is unclear.

### Pa-derived flagellin is present in the BALF of Pa-infected VAP patients

**Figure 4:**
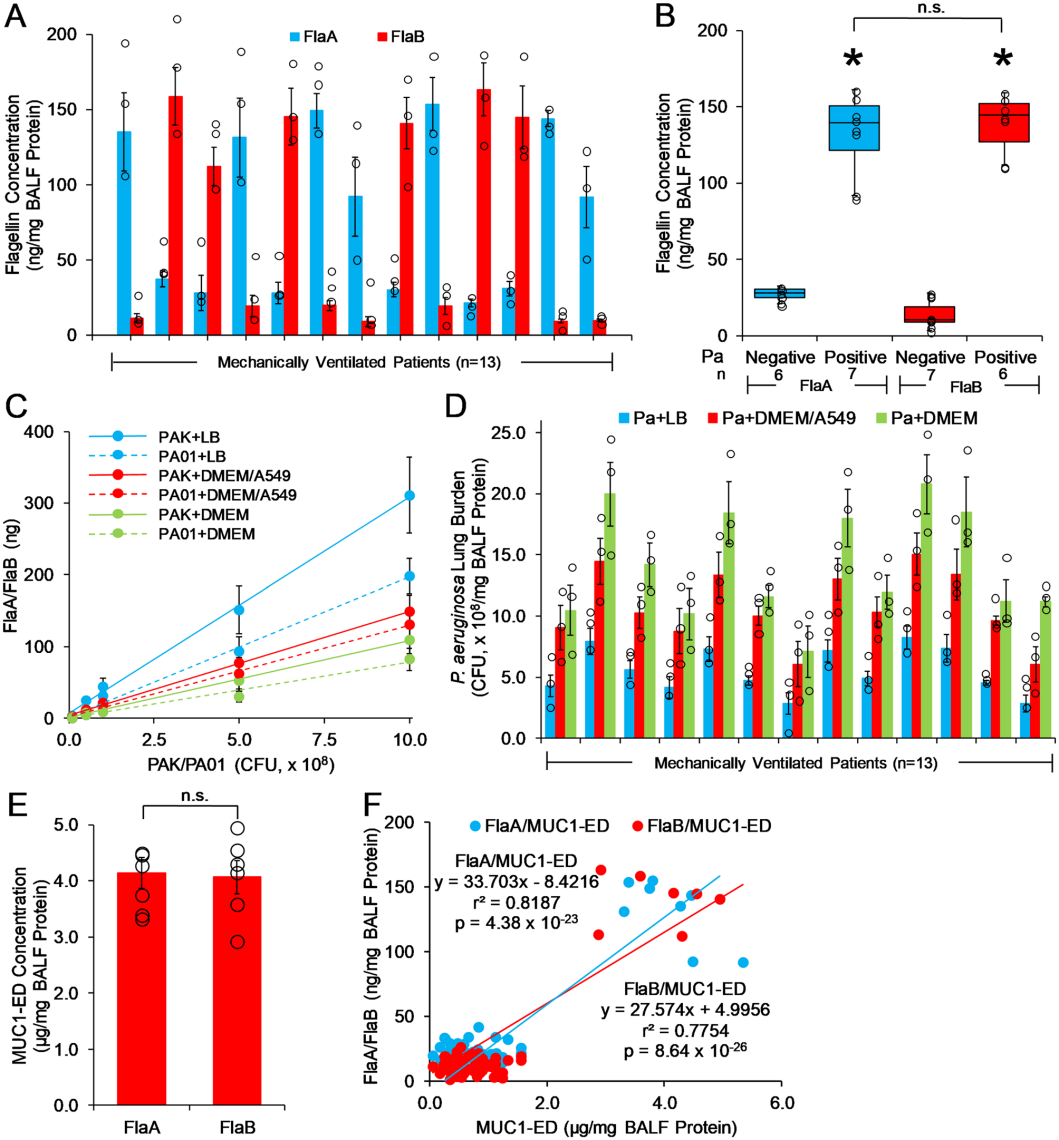
Pa-derived flagellin is present in the BALF of Pa-infected VAP patients. (**A**) Pa FlaA and FlaB flagellin levels in BALF from Pa-infected patients (n = 13) were quantified by ELISA and normalized to total BALF protein. (**B**) Box and whisker plots of the data in (**A**). *, increased MUC1-ED levels compared with FlaA-negative or FlaB-negative BALF. n.s., not significant. (**C**) Increasing CFUs of FlaA-expressing PAK or FlaB-expressing PA01 bacteria were cultured in LB medium or DMEM containing 10% FBS in the presence or absence of A549 cells. The bacteria were lysed and the lysates processed for FlaA or FlaB levels by ELISA. Standard curves were calculated by linear regression. (**D**) Pa lung burden was calculated using the flagellin levels in (**A**) with interpolation from the standard curves in (**C**). Bars represent mean ± S.E. values (n = 3). Open circles represent individual values. (**E**) MUC1-ED levels in BALFs that cultured positive for FlaA-expressing Pa (n = 7) were compared with MUC1-ED levels in BALFs that cultured positive for FlaB-expressing Pa (n = 6). Bars represent mean ± S.E. MUC1-ED levels normalized total BALF protein. Open circles represent individual values. (**F**) BALF Pa FlaA and FlaB levels in (**A**) were correlated with BALF MUC1-ED levels (Fig. 2A) and analyzed by linear regression. The results are representative of 3 independent experiments.

Flagellin-expressing Pa stimulates MUC1-ED shedding in vitro (Fig. 3), and MUC1-ED levels are increased in the BALF of mice with Pa lung infection^22^ and Pa-infected patients with VAP (Fig. 2A,B). We asked whether Pa flagellin could be detected in the BALF of Pa-infected VAP patients. FlaA and FlaB flagellin levels were quantified by ELISA and normalized to total BALF protein. The mean ± S.E. normalized FlaA level was 131.5 ± 9.1 ng/mg BALF protein (range, 91.6 to 154.9 ng/mg) in 7 of 13 VAP patients infected with FlaA-expressing Pa (Fig. 4A,B). The mean ± S.E. normalized FlaB level was 139.7 ± 6.8 ng/mg (range, 112.0 to 163.4 ng/mg) in 6 of 13 VAP patients infected with FlaB-expressing Pa (Fig. 4A,B). Only one flagellin type, either FlaA or FlaB, was present in each of the 13 patient BALFs, suggesting that only one Pa strain was responsible for each patient's infection, but not absolutely excluding coinfection with two Pa strains expressing the same flagellin type. To establish how many Pa CFUs a given flagellin level reflects, PAK and PA01 bacteria were cultured in either bacterial medium or eukaryotic tissue culture medium, in the presence or absence of A549 cells, to simulate Pa lung infection more closely, in vivo. Increasing inocula of PAK and PA01 bacteria were lysed and the lysates processed for FlaA or FlaB ELISA (Fig. 4C). Using these FlaA and FlaB standard curves, and given the comparable levels of flagellin found in laboratory and clinical Pa strains^28^, a crude estimate of the Pa lung burden for each patient was calculated. Using the FlaA/FlaB standard curves with PAK/PA01 grown in Luria–Bertani (LB) broth, a mean lung burden of 5.52 × 10^8^ CFU/mg (range, 2.82 × 10^8^ to 8.24 × 10^8^ CFU/mg) was estimated for the 13 Pa-infected VAP patients (Fig. 4D, blue bars). Using the FlaA/FlaB standard curves with PAK/PA01 grown in Dulbecco’s modified Eagle’s medium (DMEM) containing 10% fetal bovine serum (FBS) in the presence of A549 cells, a mean lung burden of 10.72 × 10^8^ CFU/mg (range, 6.03 × 10^8^ to 15.04 × 10^8^ CFU/mg) was estimated for the Pa-infected VAP patients (Fig. 4D, red bars). Using the FlaA/FlaB standard curves with PAK/PA01 grown in DMEM plus 10% FBS in the absence of A549 cells, a mean lung burden of 14.11 × 10^8^ CFU/mg (range, 7.08 × 10^8^ to 20.77 × 10^8^ CFU/mg) was estimated for the Pa-infected VAP patients (Fig. 4D, green bars). It is conceivable that the lower estimate for mean lung burden generated with Pa cultured with *vs*. without A549 cells reflects the presence of cell-associated and/or shed, Pa-targeting MUC1-ED. Next, we compared MUC1-ED levels in the BALFs that cultured positive for FlaA-expressing Pa (n = 7) to MUC1-ED levels in BALFs that cultured positive for FlaB-expressing Pa (n = 6) (Fig. 4E). No difference was detected between the two groups. Finally, each BALF FlaA or FlaB level was correlated with the BALF MUC1-ED level in the same patient for all 61 VAP patients (Fig. 4F). Linear regression analysis revealed a significant correlation between FlaA/FlaB levels and shed MUC1-ED levels (FlaA/MUC1-ED, r^2^ = 0.8187, *p* = 4.38 × 10^−23^; FlaB/MUC1-ED, r^2^ = 0.7754, *p* = 8.64 × 10^−26^). However, as with MUC1-ED levels (Supplemental Fig. 1), FlaA and FlaB levels in BALF of Pa-infected VAP patients (n = 11) did not correlate with APACHE II scores (Supplemental Fig. 3). Therefore, Pa-derived FlaA and FlaB flagellins can be detected in VAP patient BALFs, and the levels of these two flagellins each positively correlates with MUC1-ED shedding.

### Human recombinant MUC1-ED as a flagellin-targeting decoy receptor and promoter of phagocytosis

**Figure 5:**
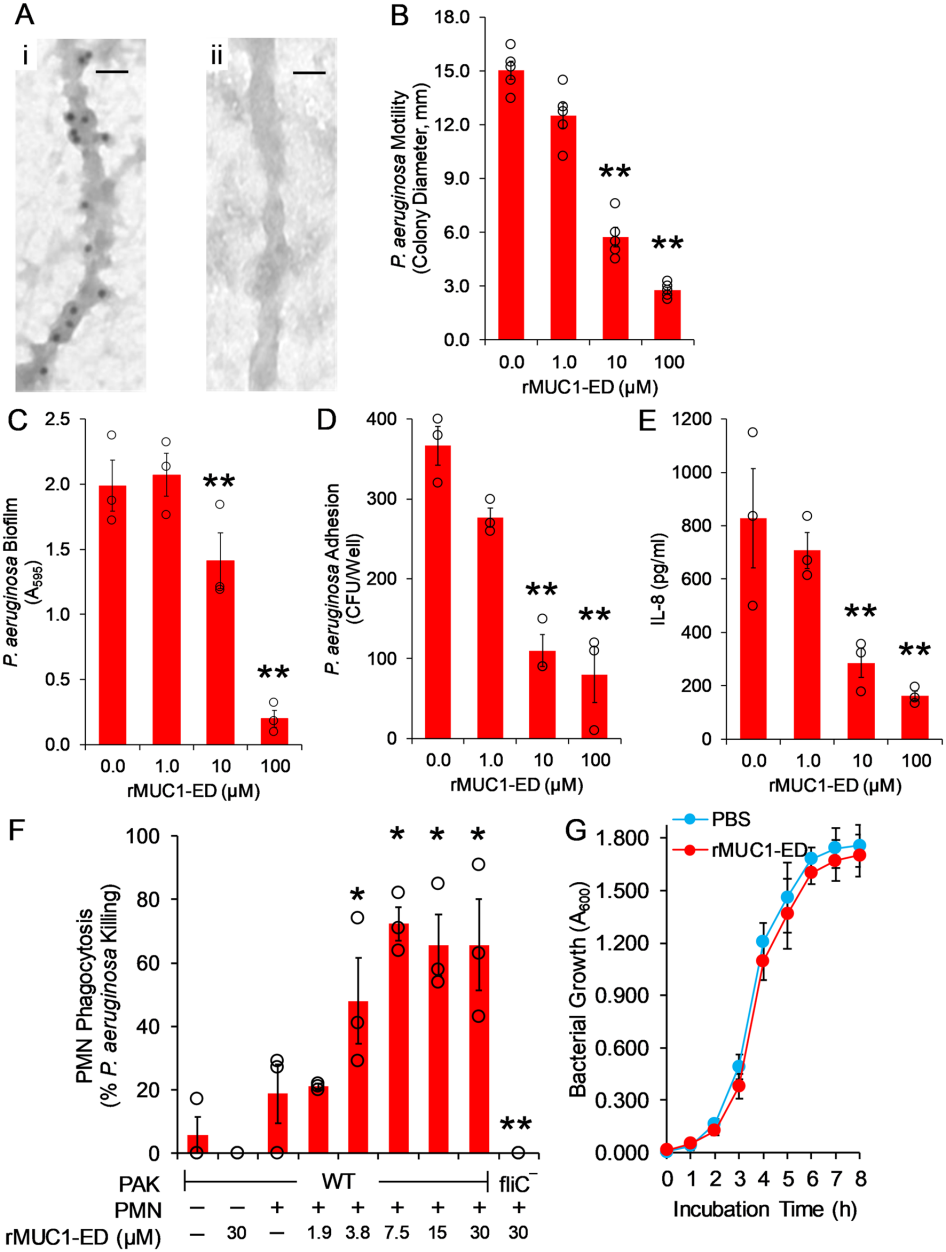
Human rMUC1-ED exhibits flagellin-targeting decoy receptor function, and enhances the PMN phagocytic response. (**A**) PAK was incubated for 1 h at 4 °C with 25 µM of human rMUC1-ED. The bacteria were washed with PBS, pH 7.4 and incubated for 1 h at 4 °C with mouse anti-MUC1-ED antibody (panel i) or nonimmune mouse IgG control (panel ii), each at 1:5,000 dilution, followed by 1 h at 4 °C with gold-labeled goat anti-mouse IgG secondary antibody at 1:10,000 dilution. Bacterial flagella were examined by transmission immunoelectron microscopy. Each section was photographed at 44,000X. Scale bar, 25 nm. (**B**, **C**) The indicated concentrations of human rMUC1-ED or the PBS vehicle control were incubated with PAK, and the bacteria were washed and assayed for (**B**) motility and (**C**) biofilm formation. (**D**, **E**) The indicated concentrations of human rMUC1-ED or the PBS vehicle control were incubated with PAK, and (**D**) the bacteria were assayed for adhesion to A549 cells and (**E**) cell culture supernatants were assayed for IL-8 levels by ELISA. (**F**) The indicated concentrations of human rMUC1-ED or the PBS vehicle control were incubated with WT-PAK or the PAK/fliC¯ flagellin-deficient strain and the bacteria were assayed for phagocytosis by PMNs. Bars represent mean ± S.E. values (n = 3). Open circles represent individual values. **, significantly decreased Pa motility, biofilm, adhesion, or IL-8 levels compared with the PBS control, or phagocytosis of PAK/fliC¯ compared with WT-PAK, at *p* < 0.05. *, significantly increased WT-PAK phagocytosis compared with the PBS control at *p* < 0.05. (**G**) PAK were cultured with 100 µM of human rMUC1-ED or the PBS vehicle, cultured over time in LB medium, and assayed for bacterial growth. Error bars represent mean ± S.E. A_600_ values (n = 3). The results are representative of 2 or 3 independent experiments.

Flagellin contributes to Pa pathogenesis through increased motility, biofilm formation, adhesion to and invasion of airway epithelia, and inflammation through activation of EC-expressed TLR5, downstream interleukin (IL)-8 production, and pulmonary leukostasis, predominantly with polymorphonuclear leukocytes (PMNs)^29–34^. In a mouse model of Pa pneumonia, endogenous murine MUC1-ED in BALF diminishes Pa motility and adhesion to airway ECs^22^. Further, using metabolic inhibitors of protein glycosylation and a nonglycosylated, *E. coli*-derived human rMUC1-ED, we demonstrated that the decoy receptor function of MUC1-ED resides within its protein backbone and not its N- or O-linked carbohydrates^22^. To further validate the utility of human rMUC1-ED as a Pa flagellin-targeting intervention, we used immunogold transmission electron microscopy (EM) to establish its binding to the intact Pa flagellum. Gold-labeled anti-mouse IgG secondary antibody immunolocalized to the Pa flagellar filament following incubation of the bacteria with human rMUC1-ED and mouse anti-MUC1-ED antibody (Fig. 5A, panel i), but not to bacteria incubated with rMUC1-ED and a nonimmune mouse IgG control (Fig. 5A, panel ii). Next, we tested human rMUC1-ED for its ability to inhibit flagellin-dependent Pa motility. Human rMUC1-ED dose-dependently inhibited Pa motility by up to 81.4%, compared with the simultaneous PBS vehicle control. (Fig. 5B). In a Pa biofilm assay where the bacteria were incubated with increasing amounts of rMUC1-ED in plastic microwells, rMUC1-ED reduced Pa biofilm formation by up to 90.5% (Fig. 5C). In a bacterial adhesion assay where Pa were cultured with A549 cells in the presence of increasing amounts of rMUC1-ED, Pa adhesion to the ECs was dose-dependently diminished by up to 78.4% (Fig. 5D). When these same ECs were incubated with Pa in the presence of the same escalating concentrations of rMUC1-ED, IL-8 levels in the cell culture supernatants were reduced by up to 80.5% (Fig. 5E). Finally, in a PMN opsonophagocytosis assay where green fluorescent protein (GFP)-expressing Pa were incubated with primary human PMNs in the presence of increasing amounts of rMUC1-ED, intracellular levels of GFP-Pa were dose-dependently elevated up to 3.87-fold compared with the simultaneous PBS control (Fig. 5F). However, human rMUC1-ED at 100 µM displayed no effect on the growth of flagellin-expressing Pa, compared with the PBS control (Fig. 5G). Thus, human rMUC1-ED, at concentrations comparable to those of MUC1-ED found in the BALF of Pa-infected patients (Fig. 2A,B) exhibits robust, flagellin-targeting decoy receptor function and enhances the PMN phagocytic response to Pa, without inhibiting bacterial replication.

## Discussion

In the current study, 21.3% of mechanically ventilated patients were infected with Pa. Pa is the second most common microorganism isolated in U.S. hospital-acquired lung infections with 90,000 cases per year^35^. In a prospective study of mechanically ventilated ICU patients across 56 sites in 11 countries, the incidence of Pa pneumonia ranged from 13.5% to 19.4%^36^. In a review of 24 studies, Pa was the most frequent isolate from ICU VAP patients, accounting for 24.4% of all microorganisms isolated^12^. Of the 13 Pa-positive patients in our study, 8 (61.5%) were identified in a chronic ventilator Comprehensive Pulmonary Rehabilitation Unit and 5 (38.5%) in an acute tertiary care medical ICU (Table 1). The increased incidence of Pa-positive patients in the chronic *vs*. acute ICU setting likely represents longer duration of intubation in the chronic ICU^7^. In fact, Pa lung infection was associated with a reduction in the number of ventilator-free days (8.2 ± 11.3 days) compared with Pa-negative patients (18.7 ± 10.0 days).

The current study supports Pa-derived flagellin as a ligand for the MUC1-ED, and suggests that flagellin engagement of the cell-associated MUC1-ED provokes MUC1-ED shedding in the airways of VAP patients. Using an in vivo experimental murine model to explore lung responsiveness to Pa, we have established flagellin-driven MUC1-ED shedding from the airway EC surface as a novel, Pa-selective host response to lung infection^22^. More specifically, purified Pa-derived flagellin dose-dependently increased MUC1-ED shedding from the murine airway epithelium into the brochoalveolar compartment. In the current study, BALF levels of the two Pa flagellin types, FlaA and FlaB, each could be tightly correlated with BALF levels of shed MUC1-ED in the same patient. Pa flagellin levels predicted MUC1-ED levels in a concentration-dependent manner. In these same patients, we found a greater than 6.0-fold increase in the mean ± S.E. MUC1-ED level in the BALF of Pa-infected VAP patients (4.01 ± 0.19 µg/mg BALF protein) compared with that observed in Pa-negative patients (0.66 ± 0.05 µg/mg) (Fig. 2A,B). No differences in BALF MUC1-ED levels were seen between patients infected with microorganisms other than Pa (0.71 ± 0.05 µg/mg) and those who were culture-negative (0.52 ± 0.08 µg/mg), indicating that the elevation of MUC1-ED levels is specific for Pa lung infection. Although *S. maltophilia* increased MUC1-ED shedding in vitro (Fig. 3C), whether BALF MUC1-ED levels are increased in patients with *S. maltophilia* lung infections is unknown. Of note, 100% of VAP patients who were Pa-positive had MUC1-ED levels ≥ 2.87 µg/mg BALF protein (range, 2.87 to 5.34 µg/mg), whereas all patients who were infected with other microorganisms or culture-negative had MUC1-ED levels ≤ 1.55 µg/mg (range, 0.05 to 1.55 µg/mg). Based on these data, 2.0 µg MUC1-ED/mg BALF protein provides a reliable diagnostic cut-off value to discriminate between Pa-infected and non-Pa-infected patients. Elevated BALF MUC1-ED levels may offer a predictive biomarker for Pa lung infection in VAP patients. The MUC1-ED ELISA is more rapid, less labor intensive, and less expensive than conventional bacterial cultures. However, the MUC1-ED ELISA identifies only Pa, and unlike bacterial cultures, does not provide antibiotic sensitivities. Further studies are required to establish whether elevated BALF MUC1-ED levels might also result from *S. maltophilia* lung infections.

In the mouse, Pa-stimulated MUC1-ED desialylation and shedding is a NEU1 sialidase-dependent process^22^. While NEU1 has historically been studied in context of its indispensable role in glycan catabolism^37^, far less is known about its role in the host response to septic processes, including Pa lung infection. We previously established NEU1 as the predominant sialidase expressed in human airway epithelial cells^23^. Silencing of NEU1 expression reduces total human lung airway EC sialidase activity by more than 70%. We detected NEU1 immunostaining in the superficial epithelium along the entire human airway, including the brush border^23^. This NEU1 expression pattern closely correlates with that known for MUC1 in these same tissues^38,39^. The MUC1-ED isolated from BALF of Pa-infected patients was desialylated (Fig. 2D). We have previously demonstrated that increased levels of soluble, desialylated MUC1-ED offer an indirect but specific measure of NEU1 catalytic activity^21,22^. We found that after siRNA-induced NEU1 silencing, MUC1-ED desialylation and shedding from Pa flagellin-exposed airway ECs is nonexistent^21^. Similarly, in a previous study, we found that NEU1-selective sialidase pharmacologic inhibition dramatically reduces MUC1-ED shedding in Pa-challenged mice^22^. These combined data indicate that NEU1 catalytic activity is increased in the lungs of Pa-infected patients. In previous coimmunoprecipitation and PNA lectin blotting studies, flagellin stimulation increased NEU1 association with and desialylation of the MUC1-ED^22^. NEU1-driven MUC1-ED desialylation unmasked not only cryptic binding sites for Pa flagellin, dramatically increasing Pa adhesion to lung ECs, but also the MUC1-ED glycine-serine protease recognition site, permitting its proteolytic cleavage and release from the airway EC surface^22^. With these results in mind, it is not surprising that desialylated MUC1-ED could not be detected in the BALF of patients who were Pa culture-negative or infected with microorganisms other than Pa (Fig. 2D, lanes 1–4).

Pa flagellin constitutes an important virulence factor through bacterial motility, adhesion to and invasion of host epithelia, and inflammatory gene expression via engagement of host TLR5^29–32^. Flagellin-dependent motility and adhesion to ECs are both prerequisites for Pa biofilm formation^33^ and flagellin is a target of opsonophagocytosis by host PMNs^34^. Host factor(s) disrupting one or more flagellin-driven processes would be predicted to protect against Pa pathogenesis. At MUC1-ED concentrations similar to those found in the BALF of Pa-infected patients, the Pa flagellin-targeting, *E. coli*-expressed human rMUC1-ED decoy receptor reduced Pa motility, adhesion to alveolar ECs and biofilm formation, as well as IL-8 production, and enhanced neutrophil-mediated Pa phagocytosis. Whether MUC1-ED bound to Pa flagellin functions as an authentic opsonin is unclear. Human rMUC1-ED exerted no measurable bacteriostatic or bactericidal activity. Through its ability to impair flagellin-driven Pa motility, MUC1-ED may interfere with Pa penetration of the mucus blanket^40^, invasion of the epithelium^23^, movement through the paracellular pathway into subepithelial tissues^41^, and/or Pa resistance to mucociliary clearance^42^. Inhibition of flagellin-dependent Pa adhesion to host airway epithelia likely decreases Pa colonization and invasion of subepithelial tissues. Disruption of the MUC1-ED-Pa flagellin receptor-ligand interaction blocks MUC1-driven downstream intracellular signaling^43^, biosynthesis of proinflammatory cytokines/chemokines^44,45^, pulmonary leukostasis^22^, and neutrophil recruitment to the bronchoalveolar compartment^46^. On the other hand, NEU1-mediated release of sialic acid residues from the highly sialylated MUC1-ED substrate may facilitate their incorporation into sialic acid-rich Pa biofilms^33^ and/or dampen complement activation^47^, thereby contributing to Pa pathogenesis.

In conclusion, MUC1-ED and Pa-derived flagellin were detected in the BALF of Pa-infected VAP patients at dramatically elevated levels compared with those found in Pa-negative patient BALF. Extending the ex vivo studies to an in vitro model of Pa infection, stimulation of human alveolar ECs with either Pa or *S. maltophilia*, but not other recognized airway pathogens, increased MUC1-ED levels in cell culture supernatants and purified rFlaA and rFlaB flagellins recapitulated the effect of intact Pa bacteria. *E. coli*-expressed, human rMUC1-ED dose-dependently inhibited Pa motility, adhesion to alveolar epithelia, biofilm formation, and IL-8 production, while at the same time, enhanced neutrophil-mediated Pa phagocytosis, without influencing bacterial growth. The current studies expand our previous observation of a flagellin-provoked, NEU1-mediated generation of a MUC1-ED decoy receptor in a mouse model of Pa pneumonia^22^ to human in vivo pathophysiology. Measurement of MUC1-ED and flagellin BALF levels offer a rapid, reliable means to identify ventilated patients with Pa pneumonia that might serve as a guide for empiric antibiotic therapy. Of note, the mechanism of action of most conventional antibiotics requires bacterial replication and cell wall remodeling^13^. It is conceivable that human rMUC1-ED, which itself does not alter bacterial replication, might be combined with antibiotics for synergistic therapy against MDR Pa.

## Methods

### Ethics statement

This study was conducted in accordance with the Declaration of Helsinki and other local statutes or regulations protecting subjects in biomedical research, and was approved by the Institutional Review Boards of the University of Maryland Baltimore (protocol numbers HP-00059183 and HP-00083805). Informed consent was obtained from all participants.

### Study setting and design

This study was conducted using BALF collected from VAP patients at the University of Maryland Medical Center (Baltimore, MD). VAP patients were admitted either to the 29-bed Medical Intensive Care Unit (MICU) or the 9-bed Critical Care Resuscitation Unit (CCRU) of the University Teaching Hospital, or received prolonged mechanical ventilation in the 14-bed Comprehensive Pulmonary Rehabilitation Unit (CPRU) of the long-term acute care University Specialty Hospital. Patient data were collected as part of routine hospital standard of care, including age, race, gender, body mass index (BMI), ICU location, ventilator-free days, antibiotics administered, chest x-ray, APACHE II score, and microbiology culture results. Bacterial identification of surveillance cultures was done at the University of Maryland Medical Center Clinical Microbiology Laboratory following standard laboratory procedures using the Vitek MS for bacterial identification and Vitek-2 system for antimicrobial susceptibilities (Biomerieux, Marcy l’Etoile, France) and interpreted according to Clinical and Laboratory Standards Institute (CLSI) criteria^48^. Clinical diagnosis of VAP was based on findings of a new pulmonary infiltrate by chest x-ray in the setting of fever, purulent sputum, leukocytosis, and diminished oxygenation^1^.

### Bronchoalveolar lavage and tracheal aspiration

BAL was performed on VAP patients undergoing standard of care diagnostic bronchoscopy. Briefly, after conscious sedation with fentanyl and midazolam, and local anesthesia with 2% lidocaine, the bronchoscope was wedged in a 3rd or 4th order bronchus, after which 125 ml of sterile pyrogen-free 0.9% NaCl was injected in 25-ml aliquots. The BALF was retrieved, pooled, filtered through sterile gauze, centrifuged at 450 xg to remove cells, and the supernatant concentrated 25-fold by ultrafiltration and stored until use at − 80 °C. Tracheal aspiration was performed using a 12 French siliconized polyvinyl chloride tracheal aspiration probe (Embramed, Brazil) as described^49^.

### Bacteria

Pa strains PA01, GFP-PA01, PAK, and PAK/fliC¯, Pa clinical strains isolated from pneumonia patients, *L. pneumophila*, *S. pneumoniae*, *H. influenzae*, *S. aureus*, and *K. pneumoniae* were previously described^22,23,50–52^. *S. maltophilia* strain 810–2 was obtained from the American Type Culture Collection (Manassas, VA). All bacteria were cultured at 37 °C in LB medium (10 mg/ml tryptone, 5.0 mg/ml yeast extract, 10 mg/ml NaCl) (Thermo Fisher Scientific, Waltham, MA), and quantified spectrophotometrically at A_600_. In selected experiments, Pa strains PA01 and PAK were cultured at 37 °C in DMEM containing 10% FBS (Hyclone Laboratories, Logan, UT) in the presence or absence of confluent monolayers of A549 human alveolar ECs (American Type Culture Collection).

### Purification of Pa and STm flagellins

An overnight culture of PA01 was centrifuged at 5000×g for 30 min, resuspended in Krebs–Ringer buffer, and incubated for 1 h at 37 °C. The bacteria were collected by centrifugation and the supernatant was filtered through a 0.22 μm pore membrane and the filtrate boiled for 20 min. The filtrate was concentrated by centrifugal ultrafiltration, adjusted to pH 6.0, and flagellin purified by sequential ion exchange chromatography using Macro-Prep High S and Macro-Prep High Q resins (Bio-Rad). Flagellin was purified from STm strain CVD1925 as described^53,54^. Pa and STm flagellins were incubated with polymyxin B-agarose (Thermo Fisher Scientific) to remove LPS, after which less than 0.1 endotoxin units/µg of protein was detected by the *Limulus* amebocyte lysate assay.

### Expression and purification Pa rFlaA and rFlaB flagellins

The DNA sequences of the *FlaA* (GenBank accession no. CP020659.1) and *FlaB* (GenBank accession no. AE004091.2) genes from PAK and PA01, respectively, were codon optimized for expression in *Escherichia coli* (GenScript, Piscataway, NJ), cloned into pUC57 and pJET1.2, respectively, and the plasmids transformed into *E. coli* NEB 5-alpha competent cells (New England BioLabs, Ipswich, MA). The *FlaA* and *FlaB* genes were amplified by PCR, cloned into pTrcHis-TOPO (Thermo Fisher Scientific), and expressed in *E. coli* BL21 competent cells (New England Biolabs) for production and purification of 6XHis-tagged rFlaA and rFlaB proteins^54^. The 6X-His-FlaA and FlaB proteins were purified on a nickel-nitrilotriacetic acid affinity column (Thermo Fisher Scientific), their identities verified by immunoblotting with anti-FlaA and anti-FlaB antibodies, and purities confirmed by a single Coomassie blue-stained protein band following SDS-PAGE.

### MUC1-ED shedding by human airway epithelia, in vitro

A549 cells (2.0 × 10^5^ cells/well) in 24-well plates were incubated for increasing times at 37 °C with 1.0 × 10^7^ CFUs/well of WT-PAK or the PAK/fliC¯ flagellin-deficient strain, or were incubated for 24 h with increasing inocula WT-PAK or PAK/fliC¯. In other experiments, A549 cells were incubated for 24 h with 1.0 × 10^7^ CFUs/well of Pa strains PA01, WT-PAK, or PAK/fliC¯, Pa clinical strains isolated from pneumonia patients, *S. maltophilia*, *L. pneumophila*, *S. pneumoniae*, *H. influenzae*, *S. aureus*, or *K. pneumoniae*. In still other experiments, A549 cells were incubated with 100 ng/well of Pa LPS (Sigma-Aldrich, St. Louis, MO), 10 µg/well of Pam_3_Cys-Ser-(Lys)_4_ (Sigma), 10 µg/well of CpG ODN 1826 (InvivoGen, San Diego, CA), 10 ng/well of Pa or STm flagellins, 10 ng/well of Pa rFlaA or rFla flagellins, or the PBS vehicle control. MUC1-ED levels in cell culture supernatants were measured by ELISA and normalized to total cell protein.

### ELISA

BALF and A549 cell culture supernatants in 96-well ELISA plates were incubated for 2 h at 22 °C with mouse anti-human MUC1-ED antibody, mouse anti-rFlaA or anti-FlaB antibodies, or mouse anti-human IL-8 antibody (Thermo Fisher Scientific). The wells were incubated for 2 h at 22 °C with 200 µg/ml of HRP-conjugated goat anti-mouse IgG secondary antibody (KPL, Gaithersburg, MD), and bound antibodies detected with tetramethylbenzidine substrate (KPL) at A_450_. Standard curves were generated using purified rMUC1-ED, rFlaA, rFlaB, and rIL-8 proteins.

### MUC1-ED immunoblotting

Equal protein aliquots of BALFs were resolved by SDS-PAGE. In order to adequately visualize the > 250 kDa MUC1-ED protein, discontinuous gel electrophoresis was performed under reducing conditions in 3% stacking/5% separating acrylamide gels containing 25 mM Tris–HCl, 190 mM glycine, 0.1% SDS, pH 8.3 until the 150 kDa prestained protein molecular weight marker reached the bottom of the gel. The resolved proteins were transferred to polyvinylidene fluoride (PVDF) membranes (Bio-Rad, Hercules, CA) and the membranes probed with rabbit anti-mouse MUC1-ED antibody, followed by HRP-conjugated goat anti-rabbit IgG secondary antibody and enhanced chemiluminescence reagents (Thermo Fisher Scientific), as described^22^.

### PNA lectin blotting of desialylated MUC1-ED

Equal protein aliquots of BALF were incubated with PNA immobilized on agarose beads (Vector Laboratories, Burlingame, CA) to selectively enrich for PNA-binding proteins and the PNA-bound proteins resolved by SDS-PAGE as described above. The resolved proteins were transferred to PVDF membranes, and the membranes processed for MUC1-ED immunoblotting as above. Asialofetuin (1.0 µg) was used as a positive control for PNA-binding proteins whereas fetuin (1.0 µg) was used as a negative control.

### Bacterial motility assay

Bacteria in mid-log phase (A_600_ = 0.5), were resuspended in LB medium, stab-inoculated into 0.3% LB agar plates, incubated overnight, and colony diameters measured as an indicator of bacterial motility, as described^22^. In other experiments, PAK were preincubated with increasing concentrations rMUC1-ED, washed, resuspended in LB medium, and analyzed for motility.

### Detection of Pa:MUC1-ED complexes

A549 cells (2.0 × 10^5^ cells/well) were cultured in DMEM containing 10% FBS in 24-well plates. The cells were incubated for 24 h at 37 °C with 1.0 × 10^8^ CFUs/well of WT-PAK or the PAK/fliC¯ flagellin-deficient strain. The bacteria were collected by centrifugation of culture supernatants at 5,000 xg for 10 min, washed 3 times with PBS, pH 7.4, and lysed. Equal protein aliquots (10 µg) of the lysates were processed for MUC1-ED immunoblotting as above.

### Expression and purification of human rMUC1-ED

Human MUC1-ED incorporating flanking *Nco*I and *EcoR*I restriction sites was amplified by PCR from a full-length MUC1-pcDNA3 plasmid^55^ and the amplicons subcloned into the pBAD/6X-His plasmid (Thermo Fisher Scientific). The plasmid was transformed into *E. coli* NEB 5-alpha competent cells (New England Biolabs), the cells were cultured to mid-log phase (A_600_ = 0.5), induced for 3 h with 0.01% arabinose, and lysed by sonication. The 6X-His epitope-tagged human rMUC1-ED was purified on a nickel-nitrilotriacetic acid affinity column, its identity verified by immunoblotting with anti-human MUC1-ED antibody, and purity confirmed by a single Coomassie blue-stained protein band following SDS-PAGE.

### Immunogold EM

Immunogold electron microscopy using a Tecnai T12 transmission electron microscope (Thermo Fisher Scientific) to detect rMUC1-ED binding to Pa flagellin was performed as previously described^56^.

### Pa biofilm assay

Overnight cultures of PAK in LB medium were adjusted to A_600_ = 0.1 in M9 medium, incubated for 48 h at 37 °C in 96-well plates with increasing concentrations rMUC1-ED, washed with deionized water, stained with 0.1% crystal violet for 10 min at 22 °C, and washed with deionized water. The crystal violet was solubilized with 30% acetic acid for 10 min at 22 °C and A_595_ measured.

### Pa adhesion to human alveolar ECs

A549 cells were cultured in DMEM containing 10% FBS. The cells were infected with adenovirus encoding NEU1 at a multiplicity of infection of 100 in 24-well plates as described^21–23^, incubated for 40 min at 37 °C with PA01 (2.0 × 10^7^ CFUs/well) in the presence of increasing concentrations of rMUC1-ED or the PBS vehicle control, and washed 3 times with PBS, pH 7.4. Bound bacteria were released with 0.05% trypsin, and CFUs quantified on LB agar plates.

### Pa opsonophagocytosis assay

An overnight culture of GFP-PA01 was inoculated into 10 ml of fresh LB medium and grown to mid-log phase (A_600_ = 0.5) in LB medium. The bacteria were centrifuged, washed, and diluted in PBS to obtain the desired number of bacteria. GFP-PA01 were incubated for 10 min at 22 °C with increasing concentrations of rMUC1-ED or the PBS vehicle control as described^57^. Approximately 1.0 × 10^6^ human PMNs for each reaction were added to the bacteria in a 96 well microtiter plate, incubated for 2 h at 37 °C, aliquots added to LB agar, and the viable colonies quantified after overnight incubation.

### Statistical analysis

For comparison of patient clinical and demographic parameters, values were expressed as mean ± S.D. All other values were expressed as mean ± S.E. The two-tailed Student’s t test, analysis of variance followed by Tukey’s post hoc analysis, and linear regression were performed using the Microsoft Excel Analysis ToolPak add-in. Statistical significance was set at p < 0.05. Power and sample size analyses were performed as described^58,59^ using our prior pilot study of BALF MUC1-ED levels in Pa-positive (n = 4) *vs*. Pa-negative (n = 11) patients^21^. The null hypothesis was defined as the mean BALF MUC1-ED level of Pa-positive patients being equal to the mean BALF MUC1-ED level of Pa-negative patients. Sample sizes for the Pa-positive group (n = 13) and Pa-negative group (n = 48) in the current study achieve 100% power to detect a difference of 3.33 µg MUC1-ED/mg BALF protein between the null hypothesis and the alternative hypothesis that the mean BALF MUC1-ED level of Pa-positive patients in the pilot study is 3.87 µg/mg BALF protein and the mean BALF MUC1-ED level of Pa-negative patients in the pilot study is 0.54 µg/mg BALF protein, with group standard deviations of 0.50 and 0.31, respectively, and with a significance level (alpha) of 0.05 using a two-sided two-sample t-test.

## Supplementary Information

Supplementary Information.
