# Supplementary material for: MUC1 ectodomain is a flagellin-targeting decoy receptor and biomarker operative during Pseudomonas aeruginosa lung infection
Source: Sci Rep. 2021 Nov 22;11:22725. doi: 10.1038/s41598-021-02242-x (PMC8608881; doi:10.1038/s41598-021-02242-x)
Supplement: Supplementary file 1 — Supplementary Information. [file 41598_2021_2242_MOESM1_ESM.docx]

**MUC1 Ectodomain is a Flagellin-Targeting Decoy Receptor and Biomarker Operative during *Pseudomonas aeruginosa* Lung Infection**

Avelino C. Verceles

Pavan Bhat

Zain Nagaria

Destiny Martin

Harsh Patel

Afua Ntem-Mensah

Sang W. Hyun

Andrea Hahn

Jean Jeudy

Alan S. Cross

Erik P. Lillehoj

Simeon E. Goldblum

**Supplemental Figure 1. MUC1-ED levels in BALF from Pa-infected VAP patients do not correlate with APACHE II scores.** MUC1-ED levels were correlated with APACHE II scores and analyzed by linear regression.

**Supplemental Figure 2. MUC1-ED levels in BALF from Pa-infected VAP patients do not correlate with MUC1-ED levels in tracheal aspirate.** (A) MUC1-ED levels in BALF and tracheal aspirates from noninfected patients (n = 7), patients infected with microorganisms other than Pa (n = 6), or Pa-infected patients (n = 2) were quantified by ELISA and normalized to total BALF or tracheal aspirate protein. Bars represent mean ± S.E. values (n = 3). (B) Linear regression analysis of the data in (A). The results are representative of 3 independent experiments.

**Supplemental Figure 3. Pa-derived FlaA and FlaB flagellin levels in BALF from Pa-infected VAP patients do not correlate with APACHE II scores.** FlaA and FlaB levels were correlated with APACHE II scores and analyzed by linear regression.

**Supplemental Figure 4. Full-length immunoblots of the blots shown in Figure 2C and Figure 2D.**

**Supplemental Figure 5. Full-length immunoblots of the blots shown in Figure 2E and Figure 3E.**
